# Development of adaptive resistance to electric pulsed field treatment in CHO cell line *in vitro*

**DOI:** 10.1038/s41598-020-66879-w

**Published:** 2020-06-19

**Authors:** Tamara Polajžer, Damijan Miklavčič

**Affiliations:** University of Ljubljana, Faculty of Electrical Engineering, Tržaška 25, 1000 Ljubljana, Slovenia

**Keywords:** Biological techniques, Cancer, Cardiology, Health care, Oncology

## Abstract

Pulsed electric field treatment has increased over the last few decades with successful translation from *in vitro* studies into different medical treatments like electrochemotherapy, irreversible electroporation for tumor and cardiac tissue ablation and gene electrotransfer for gene therapy and DNA vaccination. Pulsed electric field treatments are efficient but localized often requiring repeated applications to obtain results due to partial response and recurrence of disease. While these treatment times are several orders of magnitude lower than conventional biochemical treatment, it has been recently suggested that cells may become resistant to electroporation in repetitive treatments. In our study, we evaluate this possibility of developing adaptive resistance in cells exposed to pulsed electric field treatment over successive lifetimes. Mammalian cells were exposed to electroporation pulses for 30 generations. Every 5^th^ generation was analyzed by determining permeabilization and survival curve. No statistical difference between cells in control and cells exposed to pulsed electric field treatment was observed. We offer evidence that electroporation does not affect cells in a way that they would become less susceptible to pulsed electric field treatment. Our findings indicate pulsed electric field treatment can be used in repeated treatments with each treatment having equal efficiency to the initial treatment.

## Introduction

Electroporation or pulsed electric field (PEF) treatment can cause increase in membrane permeability and allows molecules, for which the membrane is mostly impermeable, to cross. Two distinct outcomes of electroporation can be observed and used: reversible and irreversible electroporation.

In reversible electroporation cells manage to repair the damage caused by electric pulses, *i*.e. cells survive. This has been widely used in different scientific fields for inserting molecules of interest into the cell. One of the more widely used applications is gene electro-transfer, which allows genetic manipulation. From a simple increased transfection this was developed into gene therapy^1,2^ and DNA vaccination^3,4^. While with gene therapy new genes and products are expressed within the host cell, DNA vaccination induces immune response of host organism. Combination of DNA vaccine injection and electroporation is most potent DNA delivery for subsequent immune response^5^. Increased cell membrane permeability is also being exploited in electrochemotherapy (ECT). ECT is a combination of electric pulses and chemotherapeutic drug. Due to increased cell membrane permeability chemotherapeutic drug enter the cell which potentiates the cytotoxicity of the drug^6,7^. ECT has been proved as a safe and efficient procedure for skin malignancies and now studies have been initiated to treat deep-seated tumors^8,9^.

The other possible outcome of electroporation is irreversible electroporation (IRE), where the damage to the cells is too extensive and leads to cell death. The use of irreversible electroporation as tissue ablation technique in recent years is increasing. IRE is one of the rising technologies as minimally invasive ablation treatment in interventional electrophysiology as it has several benefits over other types of ablation (*i.e*. radiofrequency or cryoablation) such as short treatment time, reduced thermal injury and selectivity or sparing of surrounding tissue. IRE can also be used for tumor and tissue ablation not amenable to surgical treatment^10,11^. In IRE treatment targeted tissue is efficiently destroyed, however, the integrity of nearby tissue structures like vessels and nerves are preserved as well as extracellular matrix^12,13^. This is also why IRE is on its way to become the leading therapy for cardiac ablation^14–16^. Other myocardial ablation therapies like radiofrequency or cryo-ablation may result in damaging untargeted tissue (e.g. phrenic nerve and esophagus)^17^. In cardiac ablation IRE, also known as pulsed field ablation (PFA), pulses are being delivered intracardially (*i.e*. inside of the heart) via specially designed catheters as minimally invasive procedure or epicardially (e.g. during heart surgery)^18^. PFA pulses use trains of mono- or biphasic pulses. Preclinical experiments show successful lesion formation in myocardium, while preserving the integrity and function of nearby structures, such as lungs, coronary arteries, phrenic nerve and esophagus^17,19–22^. First human studies showed successful and immediate electrical isolation of pulmonary vein after PFA application^23,24^.

PEF treatment including reversible (DNA vaccination, electrochemotherapy) and irreversible electroporation also stimulates sensory nerves, thus the procedure can be painful for patients^25,26^. Therefore, general anesthesia^27^, synchronization with electrocardiogram^28–30^ and administration of muscle relaxants are needed during the treatment to prevent muscle contraction, discomfort and pain due to electric pulse delivery. Electrochemotherapy and IRE are efficient and cause complete or partial response of the treated tumors/tissue which leads to prolonged survival and significant pain reduction^12,31,32^.

The outcome of the electroporation based treatment depends on pulse parameters, which may need to be adjusted to specific application and targeted tissue. While effect of different pulse parameters on cells and tissues are being addressed in *in vitro* and *in vivo* experiments, not much is known about the impact of repeated treatment, *i.e*. if cells can adapt to PEF treatment in a way that they would become less affected by the PEF treatment, *i.e*. if the treatment would become less efficient when repeated. In a recent study on human cancer cells authors suggested a development of adaptive resistance to IRE^33^. Similar question was addressed by designing experiments in bacteria for 30 generations which however showed no development of adaptive resistance to PEF treatment^34^. In our study elements from both previous studies were used for designing and conducting an experiment on developing adaptive resistance in cell exposed to PEF treatment. Mammalian cells were exposed to electroporation pulses for 30 generations over the course of four months. No development of adaptive resistance to PEF treatment was observed. We were unable to develop an electroporation resistant cell line resistant either to permeabilization (reversible electroporation) or viability (irreversible electroporation). Based on results obtained we can confirm repetitive electroporation treatment in electroporation-based therapies, like electrochemotherapy, gene electrotransfer for gene therapy or DNA vaccination and IRE as an ablation method is being as efficient as when the first time used.

## Results

**Figure 1:**
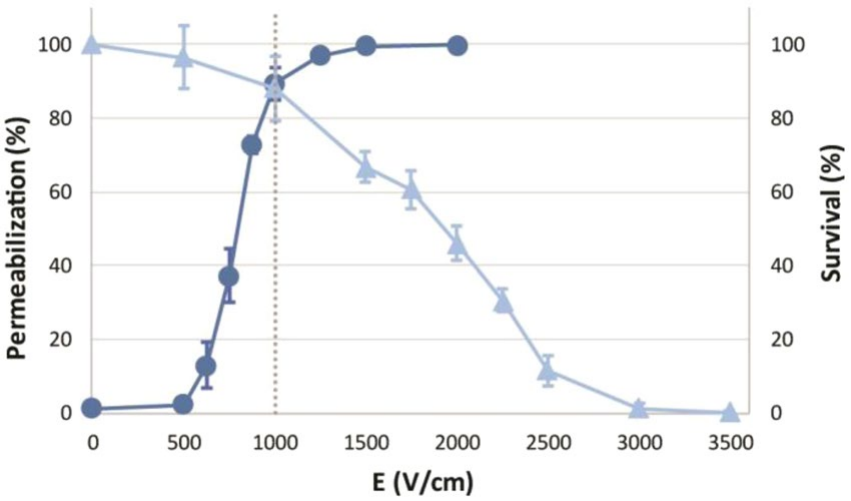
Preliminary experiments for voltage determination. Permeabilization () and survival () curve are presented. The intersection point of the curves was the selected voltage/electric field for the adaptive resistance experiment.

In preliminary experiments we determined pulse amplitude to which cells will be exposed in each generation in adaptive resistance experiment. The voltage (electric field) amplitude for the adaptive resistance experiment was selected at the intersection point of permeabilization and survival curve of 8 × 100 µs with repetition rate 1 Hz, *i.e*. 200 V (1000 V/cm) (Fig. 1). At this pulse amplitude almost all cells were permeabilized, while the survival was only mildly affected and yet enough by the standard procedure to obtain a “resistant” cell line^35^.

Graphical description of the experiment and forming of experimental groups is presented in the methods (Fig. 5). Permeabilization and survival curves (8 × 100 µs, 1 Hz, 0–600 V) were determined for 1^st^, 5^th^, 10^th^, 15^th^, 20^th^, 25^th^ and 30^th^ generation in three experimental groups: CTRL- never exposed to pulse treatment, CTRL + EP - exposed to PEF treatment only in 1^st^ generation and EP- repeatedly exposed to pulse treatment (for more detailed explanation see Materials and methods section).

**Figure 2:**
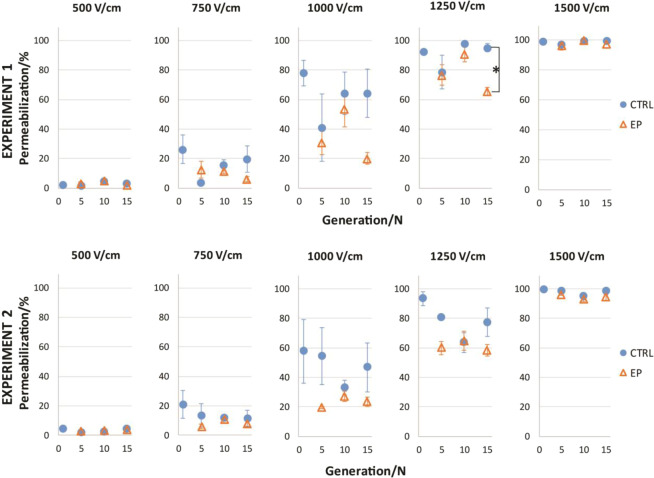
Permeabilization at different electric fields for 15 generation. Two experiments (upper and lower panels) both for 15 generations, are shown. Each experiment is presented at 5 different electric field values: 500, 750, 1000, 1250 and 1500 V/cm. Results are show an average ± SD of three technical repetitions. In each experiment generation efficiency of permeabilization of control samples (CTRL, ) was compared to samples exposed to PEF treatment/electric pulses (EP, ) in the same experiment. Statistically significant difference, with p** <** 0.05, is observed only at 1250 V/cm between CTRL and EP group of 15^th^ generation in experiment 1.

**Figure 3:**
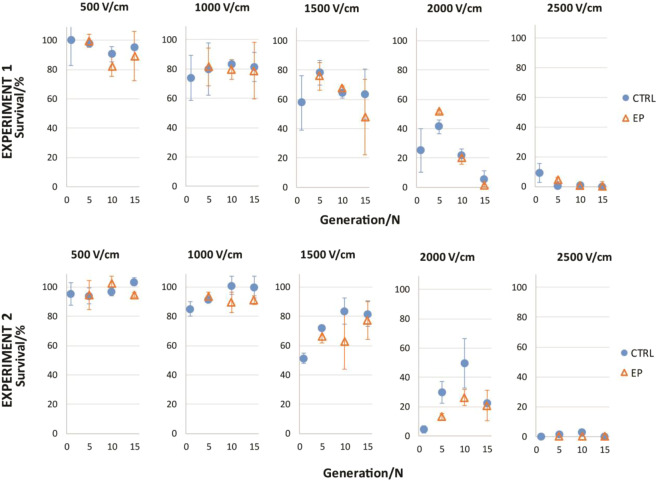
Survival at different electric fields for 15 generation. Two experiments (upper and lower panels) both for 15 generations, are shown. Each experiment is presented at 5 different electric field values: 500, 1000, 1500, 2000 and 2500 V/cm. Results are show an average ± SD of three technical repetitions. In each experiment generation efficiency of permeabilization of control samples (CTRL, ) was compared to samples exposed to PEF treatment/electric pulses (EP, ) in the same experiment. No statistically significant difference with p** <** 0.05 was observed between CTRL and EP group.

**Figure 4:**
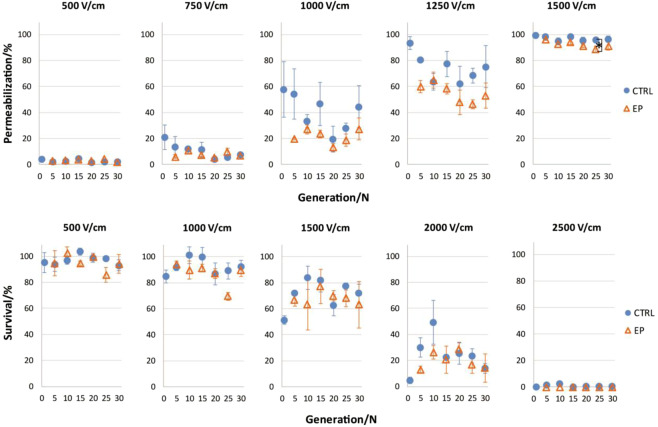
Permeabilization and survival in 30 generation. 30 generations of CTRL and EP group are shown at different electric fields. In each generation efficiency of survival of CTRL () group was compared to samples exposed to electric pulses (EP group, ). Statistically significant difference, with p** <** 0.05, is observed only at 1500 V/cm between CTRL and EP group of 25^th^ generation (note that first 15 generations are the same as those in experiment 2 from Figs. 2 and 3).

**Figure 5:**
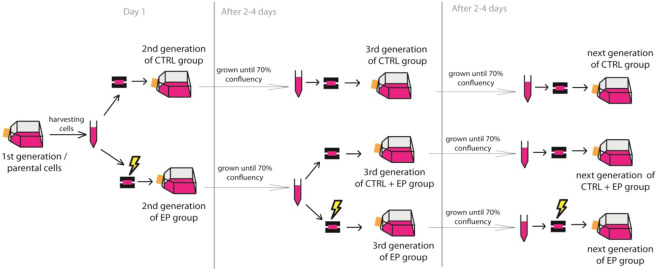
Scheme of the resistance experiment. Each generation cells were electroporated in transferred to a new growing flask. Experiment was repeated for 15 or 30 generations (N). 1^st^ generation followed by every 5^th^ generation of CTRL, CTRL + EP and EP group were analyzed for a development of adaptive resistance by preforming permeabilization and survival curve.

Over the 15/30 generation period we obtained a large amount of permeabilization and survival curves. For easier presentation we separated permeabilization and survival curves. Changes in curves at different electrical field are presented in five graphs, one for each tested point of electrical field of permeabilization and survival experiment. Rise of permeabilization curve and fall of survival curve as a function of the pulse amplitude still remains evident. This presentation allows easier comparison between CTRL and EP and between CTRL + EP and EP group at the same electric field and additionally we can compare different generations.

Every time cells were transferred to a new growing flask, they were exposed to 1000 V/cm, which caused around 80% of cells in preliminary experiment (Fig. 1) to be permeabilized. Cells were periodically exposed to this stressor, which could influence the sensitivity of cells to electroporation pulses. Looking overall at the permeabilization results obtained (Fig. 2), only one (15^th^ generation at 1250 V cm) out of 15 experimental points show statistical difference between CTRL and EP group in experiment 1. At this experimental point the slope of permeabilization curve is high and the variability between generations is large. If resistance would develop, we would expect the whole permeabilization curve (at every experimental point) to shift towards lower % values and be statistically different than control. To investigate, if this one statistically significant differences between CTRL and EP group is repeatable and not an experimental error we repeated the experiment. This time no statistical difference between CTRL and EP group (Fig. 2) was detected. After two repetitions of the experiment, no statistical differences were reproduced. Similar observations were observed between CTRL + EP and EP group. Data on CTRL + EP (cells that were only once exposed to pulse treatment) is shown in supplementary file, Fig. S1. Electroporation pulses alone do not cause/influence cells to develop adaptive resistance to cell permeabilization caused by electroporation.

In survival experiments cells were repeatedly exposed to 1000 V/cm, which in the preliminary experiment (Fig. 1) allowed around 80% of cells to survive (together 80% of permeabilization, Fig. 2). Cells were exposed to EP in every generation, which would influence the sensitivity of cells for PEF treatment, making cells more resistant to electroporation pulses, *i*.e, shifting the survival curve towards higher % values. Similar observation as in permeabilization experiment were found in viability experiments. Variation between generations is present at the slope of survival curve (Fig. 3). In the two experiments no statistically significant difference between CTRL and EP group was observed. Similar observations were observed between CTRL + EP and EP group. Data on CTRL + EP is shown in supplementary file, Fig. S2. Electroporation pulses alone do not cause cells to develop adaptive resistance to IRE.

In 15 generations of cell line, spread over the course of 2 months, we did not succeed in developing electroporation resistant cells line. On account of “short” time, we expanded the second experiment for another 15 generations, making it a total of 30 generations and prolonging the experiment to 4 months.

Only one statistically significant difference was observed in permeabilization and survival experiments (Fig. 4), however this was not present in all of the experimental points of both curves and it disappeared with a next generation. Similar results were obtained also between CTRL + EP and EP group. Data on CTRL + EP is shown in supplementary file, Fig. S3. Electroporation pulses do not cause a development of adaptive resistance in cells to PEF treatment.

## Discussion

The use of Pulsed Electric Field (PEF) treatment has increased over the last few decades. It has been successfully translated from *in vitr*o studies into clinic. Electrochemotherapy is becoming an established method for treating skin metastasis and is now moving to a deep-seated tumor^36,37^. Irreversible electroporation is on its way to become the leading therapy for hard to reach tumors^38^ and cardiac tissue ablation^39^. A promising medical field is DNA vaccination with the help of electric pulse treatment. Both reversible and irreversible electroporation are very efficient but may result in partial response or recurrent disease and thus the treatment needs to be repeated. Also in gene therapy and DNA vaccination repeatable treatment may be needed. The efficiency and impact of repeated PEF treatments is however not known. Hitherto it has not been explored if repeated PEF treatment could lead to a development of resistance to PEF treatment.

In our study, we evaluated the possibility of developing adaptive resistance to electroporation pulses. According to the available literature this was investigated only twice with different outcome *in vitro*. One experiment was performed on bacteria for 30 generations and the second experiment was on mammalian cells for 2 generations. The standard procedure to obtain a “resistant” cell line takes a few months, with low administrated doses or treatment and cells are allowed to recover in drug-free media. Produced cell line should display between two to eight-fold resistance compared to their parental cell line. In this test the sensitive parental cells are compared to the surviving daughter resistant cells by viability assays^35^. In our study we exposed mammalian cells to PEF treatment for 30 generations which lasted app. 4 months, to see if we can induce development of adaptive resistance in cells to PEF treatment. The results of our study can help to understand if PEF treatment can be used in repeated treatments in clinics.

Cells were exposed to well established and most often used electroporation pulses; 100 µs long monopolar pulses with repetition frequency 1 Hz^38,40^. Two controls were used. First control (CTRL group) represents intact parental cell line, cells that were never exposed to pulse treatment. Every time cells were just harvested and transferred to a new growing flask. This control allowed us to evaluate if pulse treatment has influenced cells to develop adaptive resistance over time. At the same time, it showed that some variably over generations is present also in untreated group. Second control (CTRL + EP) was introduced to investigate if only one exposure of cells to PEF treatment has any effect on their behavior. This happens when PEF treatment is successful and only one application is needed to achieve desired results. However, if PEF treatment results in only partial response, the treatment needs to be repeated. The baseline for this control are cells that were once exposed to PEF treatment. This control (CTRL + EP group) was first exposed to pulse treatment at the beginning of the experiment and then it was just continually transfer to a new growing flask in an absence of pulse treatment. Two controls in experiment allowed us to assess, if repetitive exposure to PEF treatment leads to any changes in cell behavior compared to cells that were never exposed to PEF treatment and cells with only one exposure to PEF treatment (cells that had only partial response to the first treatment).

In our experiment 1^st^ generation followed by every 5^th^ generation was being analyzed by determining permeabilization and survival curve. We would expect a shift, *i.e*. higher electrical field would be needed for permeabilization and survival curve of EP group, in comparison to control groups, if cells exposed to pulsed electric field treatment develop adaptive resistance (become resistant) to the electroporation pulses. Furthermore, we would expect the shift between EP and control groups to become bigger with each generation.

Looking overall at our result only in two data points statistical difference between CTRL and EP group and one between CTRL + EP and EP group were detected, which, however, disappeared with the next generations and with the repetition of the experiment. We thus assumed these differences are most probably a result of experimental errors and can as such be considered random. No repeatable statistical difference between EP and control groups and no visible shift in permeabilization or survival curves were observed. Electric field for no (0%) and maximum/full permeabilization (100%) and cell death stays the same even after 30 generations. Nevertheless, some variations between generations within the same experimental group (within CTRL group, within CTRL + EP group and within EP group) can be observed at intermediate electric fields/experimental points which however do not provide evidence of cells developing adaptive resistance. According to our results PEF treatment can thus be used for repeated treatments with similar efficiency as the initial treatment.

Our findings can hardly be compared to the results of previously reported study^33^ where a small number of generations (only 2: 1^st^ and 2^nd^ generation) was used. A small number of generations used invalidates the conclusion of authors regarding adaptive resistance to PEF treatment being developed. Namely, according to standard procedure obtaining a “resistant” cell line takes a few months, while the study in^33^ on adaptive resistance development due to electrical pulses based on 2 generation took only 5 days. Such a short time is not sufficient to develop a resistant cell line. In our experiment we observed a statistical difference between CTRL and EP group at 5^th^ generation, but with the next generation this difference disappeared, and the difference was attributed to experimental error. Also, experiments in these two studies were performed on different cell lines; in^33^ experiments were performed using human pancreas cancer cells, whereas in our experiment “healthy” cells from hamster ovaries were used so direct comparison is not necessarily adequate.

Even though our result show that PEF treatment can be used as safe and efficient in repeated treatments, we need to recognize some limitations of our study. Since the standard procedure to obtain a resistant cell line is conducted with low administrated doses of chemicals or treatment, we believe affecting 80% of cell viability (*i.e*. 20% cell kill) should be sufficient enough to cause a development of resistant cell line. At the same time, chosen threshold caused 80% in permeabilization assay, which from permeabilization perspective was actually a high administrated dose. Nevertheless, no development of adaptive resistance to PEF was detected in permeabilization. We believe that same cell behavior would we observed in development of adaptive resistance to PEF in survival assay, even if the used electric field would be higher. However, it would be interesting to test, if adaptive resistance to PEF in survival assay would change, if experiment was performed with PEF treatment causing higher, e.g. 80% cell kill. The study was performed only on one cell line, *i.e*. CHO cell line, which are genetically stable and are considered as non-cancer cells. For more reliable results, different cancer and non-cancer cell should be used. However, when working with cancer cell line, number of passages (generation) is limited between 10–20, depending on cell line, as low passage reflects the characteristics of the primary tumor more closely and the malignancy kept^41,42^. Such experiments would be shorter than the one performed in this study, but sufficient enough to see, if cancer cell behavior is different form non-cancer cell. If the adaptive resistance would be obtained *in vitro* further *in vivo* experiments would be necessary.

## Conclusion

In our study the efficiency and the effect of repeated PEF treatment was investigated *in vitro* on CHO cells. Our results show that electroporation does not affect cells in a way that they would become less susceptible to PEF treatment, *i.e*. that cells would develop adaptive resistance for reversible or irreversible electroporation. Our findings indicate PEF treatment can be used for repeated treatments, if the initial treatment resulted in only partial response or recurrence of disease.

## Methods

### Pulses

Eight 100 µs long monopolar pulses with repetition frequency 1 Hz of 100–600 V (0.5–3 kV/cm) were applied by the laboratory prototype pulse generator (University of Ljubljana), based on H-bridge digital amplifier with 1 kV MOSFETs (DE275-102N06A, IXYS, USA)^43^. Delivered current and voltage were measured by the oscilloscope Wavesurfer 422, 200 MHz, the current probe CP030 and the differential probe ADP305 (all from LeCroy, USA) (recording not shown). Cells in suspension were electroporated between stainless steel 304 plate electrodes with 2 mm interelectrode distance (voltage-to-distance ratio: kV/cm).

### Cells

Chinese hamster ovary (CHO-K1, European Collection of Authenticated Cell Cultures) (negative for mycroplasma) were grown in HAM F-12 growth medium (PAA, Austria) in culture flasks (TPP, Switzerland) in an incubator (Kambič, Slovenia) at 37 °C with a humidified 5% CO_2_. The growth medium was supplemented with 10% fetal bovine serum (FBS) (Sigma-Aldrich, Germany), L-glutamine (StemCell, Canada), antibiotics penicillin/streptomycin (PAA, Austria) and gentamycin (Sigma-Aldrich, Germany) (*i.e*., complete HAM-F12). When 70% confluency was reached, cells were detached by 10x trypsin-EDTA (PAA, Austria), diluted 1:9 in Hank’s basal salt solution (StemCell, Canada) and incubated at 37 °C for 2 minutes. Trypsin was inactivated by addition of fresh complete HAM F-12. Sample was centrifuged at 180 g and 22 °C for 5 minutes. Old media was removed, and the cell pellet was re-suspended in growth medium (complete HAM-F12) at the cell density 2 × 10^6^ cells/ml.

### Permeabilization assay

Electropermeabilization of cells can be quantified by penetration of a membrane-impermeable fluorescent agent like propidium iodide (PI)^44^. Binding of PI to nucleic acid enhances its fluorescence, so it can easily be detected. Before electric pulses were applied, cells suspension was mixed with propidium iodide (PI, Life Technologies) to a final concentration of 100 µg/ml. 60 µl of the sample was transferred between electrodes, and electric pulses were applied. Afterwards the sample was transferred to a 1.5 ml tube and incubated at room temperature for three minutes. 150 µl of growth medium was added to obtain a high-enough volume for measurement. The uptake of PI was detected with the flow cytometer (Attune NxT; Life Technologies, Carlsbad, CA, USA). Samples were excited with a blue laser at 488 nm and emitted fluorescence was detected through a 574/26 nm band-pass filter. 10,000 events were obtained, and data were analyzed using the Attune Nxt software. In analysis single cells were separated from debris and clusters and the percentage of PI permeabilized cells was obtained from PI fluorescence intensity histogram by gating permeabilized cells from non-permeabilized.

### Viability assay

The MTS Assay is a colorimetric method for determining the number of viable cells^45^. 60 µl of the sample was transferred between electrodes, and electric pulses were applied. After pulse application, 40 µl cell suspension was diluted in full HAM-F12 growth media to obtain cell density 2 × 10^4^ cells/100 µl. 100 µl of cell suspension was then transferred (in three technical repetitions) to wells in the 96-well plate (TPP, Switzerland) and incubated at 37 °C and humidified 5% CO_2_ atmosphere. MTS assay (CellTiter 96 AQueous One Solution Cell Proliferation Assay, Promega, USA) was used to assess cell viability 24 hours after electric pulses were applied. According to manufacturer’s instructions 20 µl of MTS tetrazolium compound was added to the samples, and the 96-well plate was returned to the incubator for 2 hours. The absorbance of formazan (reduced MTS tetrazolium compound) was measured with a spectrofluorometer (Tecan Infinite M200, Tecan, Austria) at 490 nm. Percentage of viable cells was calculated by subtracting the background and normalizing the absorbance of the samples to the absorbance of the sham control (0 V/cm).

### Development of adaptive resistance experiment

In preliminary experiments we determined amplitude of voltage/electric field at which high membrane permeabilization and cell survival at the same time is achieved.

Parental cells (1^st^ generation) were detached and resuspended in complete HAM F-12 growth media at the density of 2 × 10^6^ cells/ml. Parental cells were split to two groups: one was used as a control in the absence of any pulse treatment – control or parental cells, never exposed to pulse treatment (CTRL group), and the other was repeatedly exposed to pulse treatment to become “resistant” cell line by exposing cells to pulse treatment (EP group). 60 µl samples were placed between plate electrodes and pulses were delivered (8 × 100 µs, 1 Hz, 200 V). Control cells were subjected to the same procedure as the exposed sample in absence of pulses, *i.e*. 0 V/cm amplitude. Afterwards cells were transferred in new culture growing flask and placed in an incubator until a new generation of cells grew to obtain 70% of confluency was reached (2–4 days).

Cells from the second generation (CTRL and EP group) cells were harvested and resuspended incomplete HAM F-12 growth medium to the density of 2 × 10^6^ cells/ml. CTRL group was again transferred between the electrodes (in the absence of pulse treatment) and then transferred to the new growing flask. EP group was split in two parts. One part was exposed to pulse treatment and then transferred to the new growing flask. The other part became a control, which was once exposed to electric pulses (CTRL + EP). Cells were transferred between the electrodes (in the absence of pulse treatment) and then transferred to the new growing flask. Cell were incubated until a new generation of cell grew.

Cells from the third generation (CTRL, CTRL + EP and EP group) were harvested and resuspended in complete HAM F-12 growth medium to the density of 2 × 10^6^ cells/ml. Cells from control groups (CTRL and CTRL + EP group) were transferred between the electrodes (in the absence of pulse treatment) and then transferred to the new growing flask. Cells from EP group were exposed to pulse treatment) and then transferred to the new growing flask. Cell were incubated until a new generation of cells grew. This procedure was repeated until a wanted generation for all of the groups was obtained.

Graphical description of the experiment is presented in Fig. 5. This process/experiment was repeated twice for 15 generations. Second experiment was prolonged to 30 generations. Three technical replications were made for each group in each experiment.

The first, followed by every fifth, generation in EP and control groups (5th, 10th, 15th, 20th, 25th and 30th) were analyzed. In every fifth generation permeabilization and survival curves (typical for electroporation assays) were determined (8 × 100 µs, 1 Hz, 0–600 V). If cells exposed to electroporation pulses (EP group) would develop adaptive resistance to the PEF treatment, we would expect a shift in permeabilization and/or survival curve in comparison to control groups.

### Statistical analysis

Statistical analysis was performed using SigmaPlot 11.0 (Systat Software, USA). The results are shown as mean ± SD. Statistically significant differences (*p < 0.05) were determined by repeated measures ANOVA test, followed by multiple comparison by Hold-Sidak method.

## Supplementary information

Supplementary information.
