# Supplementary material for: Development of adaptive resistance to electric pulsed field treatment in CHO cell line in vitro
Source: Sci Rep. 2020 Jun 19;10:9988. doi: 10.1038/s41598-020-66879-w (PMC7305184; doi:10.1038/s41598-020-66879-w)
Supplement: Supplementary file 1 — Supplementary information. [file 41598_2020_66879_MOESM1_ESM.docx]

Supplementary: Development of adaptive resistance to electric pulsed field treatment in CHO cell line *in vitro*

Tamara Polajžer, Damijan Miklavčič


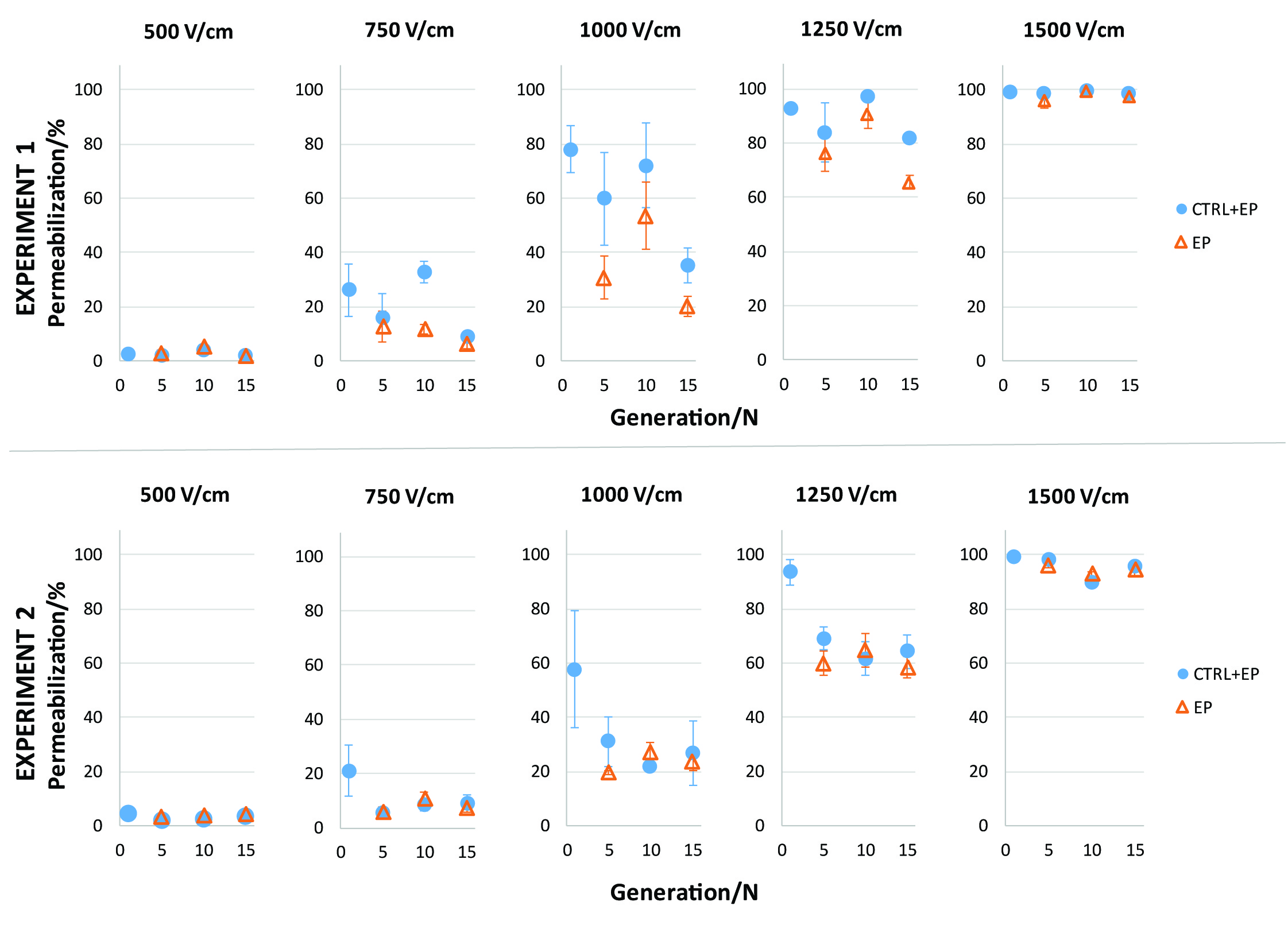


Figure S1: Permeabilization at different electric fields for 15 generation. Two experiments (upper and lower panels), both for 15 generations, are shown. Each experiment is presented at 5 different electric field values: 500, 750, 1000, 1250 and 1500 V/cm). Results are show an average ± SD of 3 technical repetitions. In each generation efficiency of permeabilization of control samples (CTRL+EP, ) was compared to samples exposed to PEF treatment/electric pulses (EP, Δ). No statistically significant difference with p<0.05 was observed between CTRL and EP group.


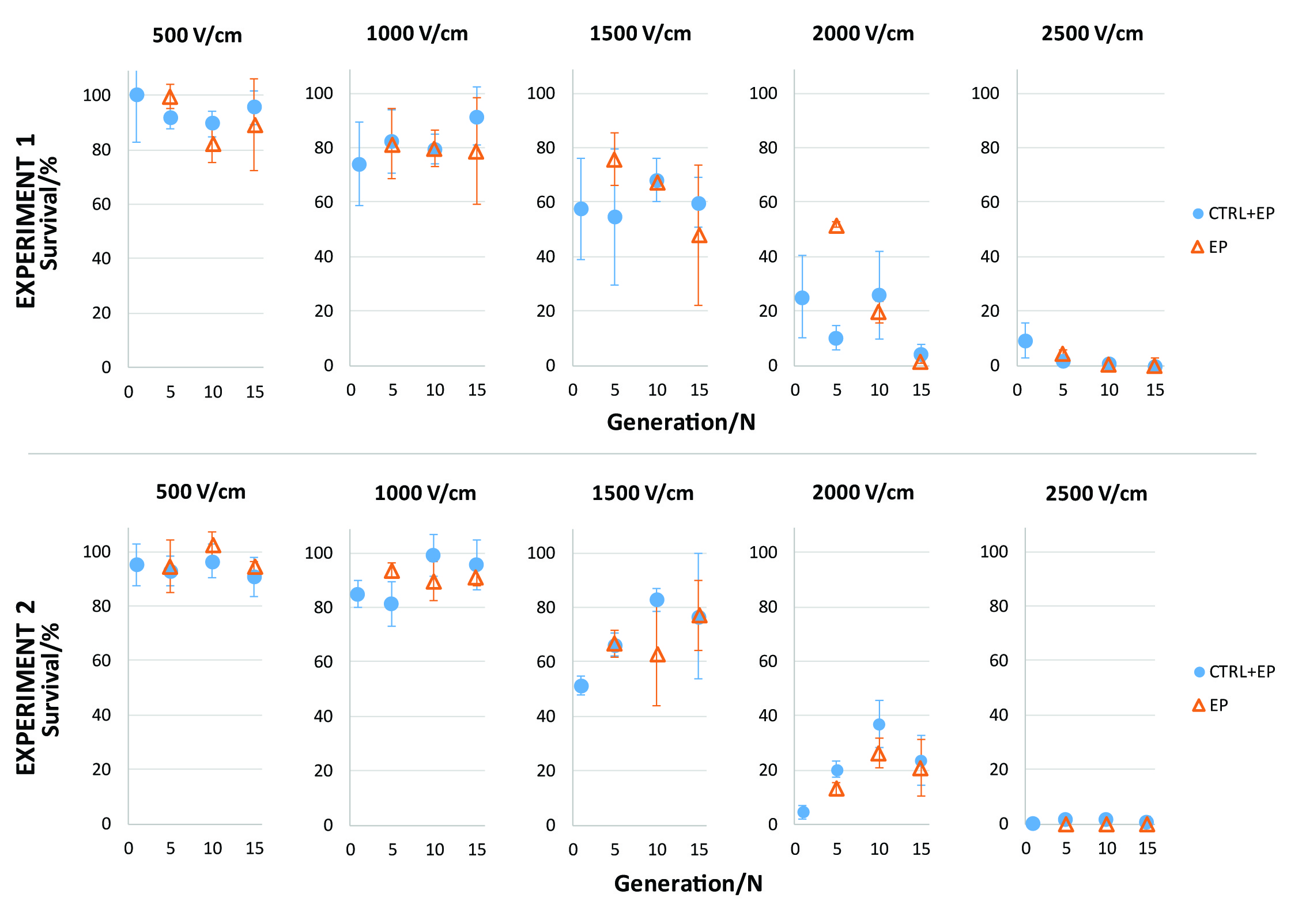


Figure S2: Survival at different electric fields for 15 generation. Two experiments (upper and lower panels), both for 15 generations, are shown. Each experiment is presented at 5 different electric field values: 500, 1000, 1500, 2000 and 2500 V/cm). In each generation efficiency of survival of control samples (CTRL+EP, ) was compared to samples exposed to electric pulses (EP, Δ ). No statistically significant difference with p<0.05 was observed between CTRL and EP group.


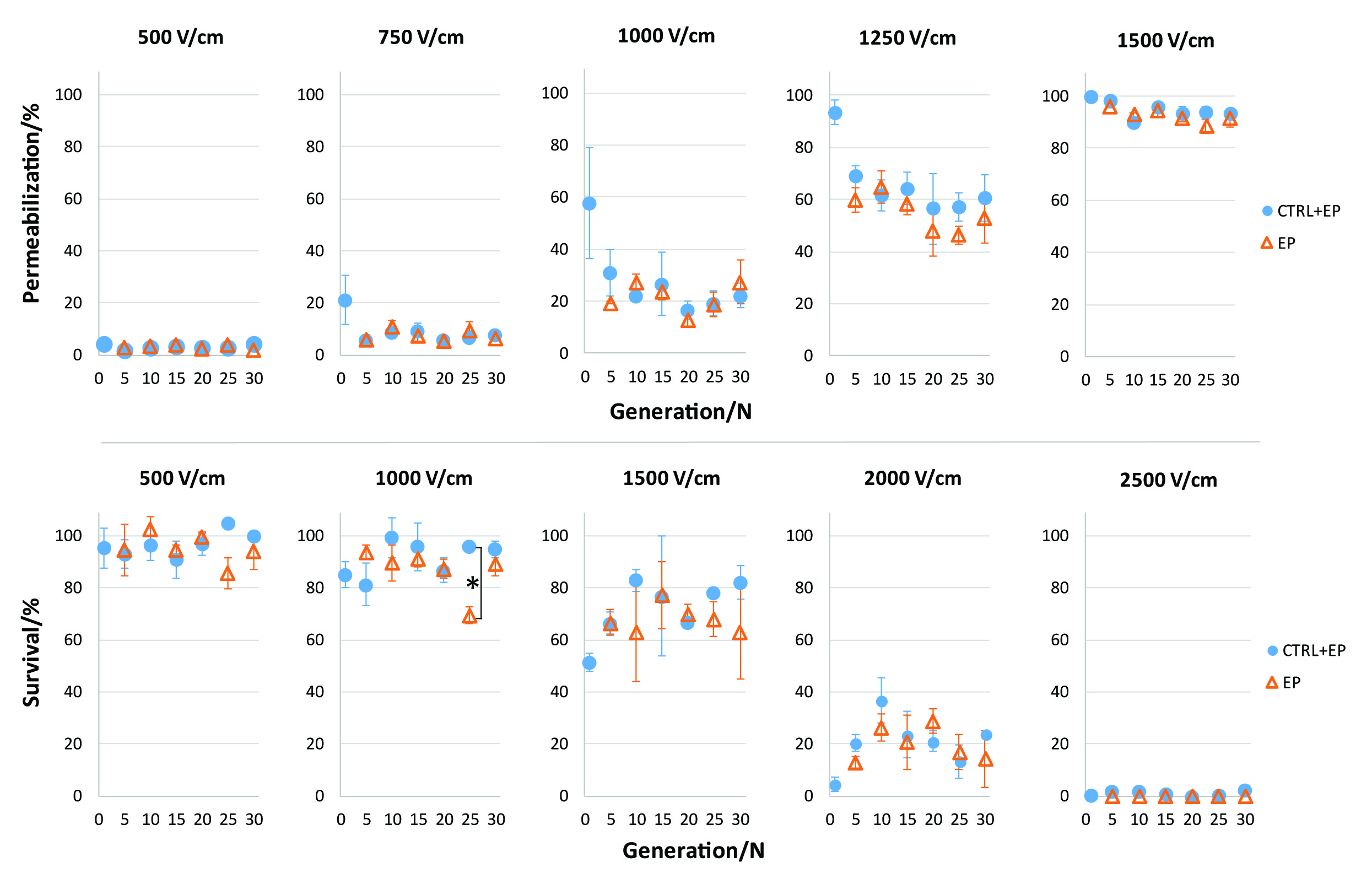


Figure S3: Permeabilization and survival in 30 generations. 30 generations of CTRL+EP () and EP group (Δ) are shown at different electric fields. In each generation efficiency of survival of CTRL+EP group was compared to samples exposed to electric pulses (EP group). Statistically significant difference, with p<0.05, is observed only at 1000 V/cm between CTRL and EP group of 25^th^ generation (note that first 15 generations are the same as those in experiment 2 from figures S1 and S2). This difference however “disappeared” in the 30^th^ generation tested.
